# Bioavailability of Zinc in Wistar Rats Fed with Rice Fortified with Zinc Oxide

**DOI:** 10.3390/nu6062279

**Published:** 2014-06-13

**Authors:** Ceres Mattos Della Lucia, Laura Luiza Menezes Santos, Kellen Cristina da Cruz Rodrigues, Vivian Cristina da Cruz Rodrigues, Hércia Stampini Duarte Martino, Helena Maria Pinheiro Sant’Ana

**Affiliations:** Department of Nutrition and Health, Federal University of Viçosa, Avenida Purdue, s/n, Campus Universitário, Viçosa-MG 36.570-900, Brazil; E-Mails: laura.santos@ufv.br (L.L.M.S.); kellen.rodrigues@ufv.br (K.C.C.R.); vivian.cruz@ufv.br (V.C.C.R.); hercia@ufv.br (H.S.D.M.); helena.santana@ufv.br (H.M.P.S)

**Keywords:** zinc carbonate, bioassay, micronutrient deficiency

## Abstract

The study of zinc bioavailability in foods is important because this mineral intake does not meet the recommended doses for some population groups. Also, the presence of dietary factors that reduce zinc absorption contributes to its deficiency. Rice fortified with micronutrients (Ultra Rice^®^) is a viable alternative for fortification since this cereal is already inserted into the population habit. The aim of this study was to evaluate the bioavailability of zinc (Zn) in rice fortified with zinc oxide. During 42 days, rats were divided into four groups and fed with diets containing two different sources of Zn (test diet: UR^®^ fortified with zinc oxide, or control diet: zinc carbonate (ZnCO_3_)), supplying 50% or 100%, respectively, of the recommendations of this mineral for animals. Weight gain, food intake, feed efficiency ratio, weight, thickness and length of femur; retention of zinc, calcium (Ca) and magnesium (Mg) in the femur and the concentrations of Zn in femur, plasma and erythrocytes were evaluated. Control diet showed higher weight gain, feed efficiency ratio, retention of Zn and Zn concentration in the femur (*p* < 0.05). However, no differences were observed (*p* > 0.05) for dietary intake, length and thickness of the femur, erythrocyte and plasmatic Zn between groups. Although rice fortified with zinc oxide showed a lower bioavailability compared to ZnCO_3_, this food can be a viable alternative to be used as a vehicle for fortification.

## 1. Introduction

Numerous studies have shown the increasing number of chronic non-communicable diseases worldwide, whose causes point primarily to lifestyle as the main etiological factor. In general terms, the Western diet is rich in sugars, saturated and trans fats, lacks dietary fiber and regarding micronutrients, zinc (Zn) is among the most deficient [1]. Studies in Latin American countries and the United Stated showed that average Zn intake varies between 50% and 80% of that recommended, regardless of age, gender and race [2].

Although there are few studies in the literature that evaluate the prevalence of zinc deficiency in populations of Brazil and worldwide, it is suggested that it is as frequent as that of iron. Deficiencies can coexist, because of the similarity of food sources as well as factors that impede absorption of these minerals [3]. Deficiency of Zn is associated, among other alterations, to immune system dysfunction, stunted growth and high risk of morbidities such as diarrhea, respiratory infections and chronic non-communicable diseases [4].

Zinc participates in several functions in the body, explained in part by its structural role in the formation of enzymes that act as endogenous antioxidants, such as superoxide dismutase, also acting in the stabilization of protein domains that interact with DNA [5].

Zinc absorption in mixed diets is approximately 30% and is influenced by the solubility of Zn compounds in the diet and by its competition with other minerals for transporters or intestinal uptake sites, among other factors [6]. The molar ratio between phytate and zinc in foods or in a mixed diet is a useful indicator of phytic acid’s effect in reducing the absorption of zinc. In phytate: at zinc molar ratios exceeding the range of 6–10, zinc absorption begins to decrease; at ratios greater than 15, absorption is typically less than 15%. The effect of phytate, however, is modified by the amount of protein consumed. Animal protein consumption increases zinc absorption in a diet rich in phytate [7].

Assessment of the nutritional status of zinc of individuals is complicated by the fact that no generally accepted, sensitive and specific biomarker of zinc status exists [8]. Although plasma zinc concentrations decrease within several weeks of the introduction of a diet containing a restricted amount of zinc [9], plasma zinc concentrations are generally maintained within the normal range with small or moderate reductions in zinc intake. Other factors unrelated to the nutritional status of zinc, such as recent meals, time of day, infection and pregnancy can also affect plasma zinc concentrations [8]. Thus, the serum zinc concentration may not always be a reliable indicator of an individual’s true zinc status. Other tools used for assessing zinc’s bioavailability are erythrocyte zinc, food efficiency ratio (FER) and the content of zinc in the bone [10].

Study of the zinc bioavailability in foods is important because the quantities consumed do not meet the recommended doses for some population groups. The presence of factors that reduce absorption contributes to the development of deficiency [11].

Food fortification is in one of the most efficient strategies to reduce micronutrient deficiency and it is socially acceptable, requiring no change in eating habits and does not alter the characteristics of foods [12].

The Ultra Rice^®^ (UR*^®^*, Adorela, Indaiatuba, Sao Paulo, Brazil) fortification technology consists of transforming broken rice grains into rice flour, which is combined with fortifying nutrients, and remolded into rice grains with the same size, shape and texture of polished rice. The levels of fortification agents can be concentrated in these grains, so that they may be mixed with polished rice at a ratio of 1:50 to 1:200 [13].

This study is based on the assumption that the UR^®^ fortified with Zn, in the form of zinc oxide, shows good bioavailability in the food matrix of rice supplemented with other fortification agents (such as ferric pyrophosphate and thiamine mononitrate, for example, used in fortification of rice). However, the widespread utilization of fortified foods as a strategy for control of vitamin and mineral deficiency indicates the need to investigate possible interactions between micronutrients [14]. Because studies evaluating the bioavailability of Zn in UR^®^ are nonexistent to date, and in an attempt to provide data regarding the best characterization of this product, this study sought to evaluate the bioavailability of Zn in rats fed UR^®^ fortified with zinc oxide.

## 2. Experimental Section

The present study was developed in the Laboratory of Experimental Nutrition, Department of Nutrition and Health and in the Laboratory of Atomic Absorption Spectrometry, Department of Soil Science, Federal University of Viçosa.

The study was conducted according to Brazilian Standards for Animal Experimentation and was approved by the Ethics Committee on Animal Research of the Federal University of Viçosa (Process No. 33/2011).

### 2.1. Raw Material

Rice grains extruded from rice flour were used (Ultra Rice^®^—UR^®^). They were produced and provided by a manufacturer of pasta after authorization by the Program for Appropriate Technology in Health (PATH). The grains contained iron (in the form of micronized ferric pyrophosphate), zinc (in the form of zinc oxide), thiamine (in the form of thiamine mononitrate) and folic acid.

### 2.2. Experimental Diets

Composition of the experimental diets was based on the AIN-93G diet [15] (Table 1) with the mineral mixture containing no Zn. Diets were appropriately adjusted to provide 15 or 30 mg Zn/kg of the diet, equivalent to 50% or 100% of the recommendation of this mineral for the animals, obtained from zinc oxide present in UR^®^ for the groups R15 and RA30 (since this fortified rice was formulated with zinc oxide as a source of zinc) and zinc carbonate (ZnCO_3_ = 521 mg Zn/g) (the zinc salt recommended in AIN-93G) for the groups C15 and C30. The groups were categorized as follows: R15: Source of Zn—Rice fortified with zinc oxide, Zn 50%; R30: Source of Zn—Rice fortified with zinc oxide, Zn 100%, C15: Source of Zn—zinc carbonate, Zn 50%, C30: Source of Zn—zinc carbonate, Zn 100%.

**Table 1:** Composition of experimental diets (g/100 g mixture) used in the essay of bioavailability of zinc.

Components *	Test Diets		Control Diet	
	R15 (g/100 g)	R30 (g/100 g)	C15 (g/100 g)	C30 (g/100 g)
ZnCO_3_ (mg)	-	-	0.002876	0.005752
Dietary iron (ferric pyrophosphate)	0.0059	0.0059	0.0059	0.0059
*Ultra Rice^®^* ^a^	1.14	2.28	-	-
Albumin **	19.91	19.83	20.00	20.00
Maltodextrin **	13.20	13.20	13.20	13.20
Sucrose **	10.00	10.00	10.00	10.00
Soybean oil **	6.94	6.87	7.00	7.00
Fiber (microfine cellulose) **	4.98	4.98	5.00	5.00
Mineral mix without zinc **	3.50	3.50	3.50	3.50
Vitamin mix **	1.00	1.00	1.00	1.00
l-cystine **	0.30	0.30	0.30	0.30
Choline bitartrate **	0.25	0.25	0.25	0.25
Corn starch **	38.77	37.79	39.75	39.75
Caloric density (kcal/g)	3.69	3.69	3.78	3.78

* According to Reeves *et al.* (1993) [15]; ** Rhoster, Industry and Commerce, R15: Source of Zn:Rice fortified with zinc oxide, Zn 50%; R30: Source of Zn:Rice fortified with zinc oxide, Zn 100%; C15: Source of Zn:Zn carbonate, Zn 50%; C30: Source of Zn:Zn carbonate, Zn 100%; ^a^ 0.13% of zinc.

The ingredients of the diets were individually weighed and mixed in a semi-industrial mixer (Lieme^®^, São Paulo, Brazil), at low speed for 20 min. The finished diets were packaged in polyethylene bags and stored at 10 °C.

### 2.3. Determination of Zn in UR^®^ and in the Experimental Diets

For determination of Zn, digestion of the sample was performed, in triplicate, by weighing 1 g of UR^®^ or of the experimental diets in a digestion tube, followed by addition of 10 mL of concentrated HNO_3_. The digester block was turned on to 80 °C and the temperature was gradually increased up to 160 °C. After the first 8 h of digestion, 5 mL of HNO_3_ were added. After completion of digestion, the tube contents were quantitatively transferred to a 50 mL demineralized volumetric flask. Then, the sample was vortexed and the volume of the flask was completed with deionized water. Reading was performed using an atomic absorption spectrophotometer (Perkin-Elmer Optima 3300 DV, Norwalk, CT, USA). The glassware and utensils used in determination of the mineral content and in the biological assays were demineralized, using a 10% HNO3 solution, in which they remained for 24 h to be subsequently rinsed with deionized water.

### 2.4. Determination of the Bioavailability of Zn in Vivo

Forty male rats (*Rattus norvegicus*, variety *albinus*, class *Rodentia*), of the lineage Wistar, recently weaned, with body weight between 67.7 g and 94.8 g, obtained from the Central Animal Laboratory of the Federal University of Viçosa, were used in the experiment. The experimental groups were balanced in terms of weight at baseline because the starting weight can influence the subsequent weight gain. They were maintained in individual stainless steel cages, in an environment with controlled temperature (22 ± 2 °C) and light, submitted to a 12 h light-dark cycle.

The animals were divided into four groups with 10 animals each and were maintained on their respective diets for 42 days, time during which they received deionized water *ad libitum* and ingestion of the controlled diet varied between 16 and 17 g daily. Weight of the animals was monitored weekly, as well as food intake, thus calculating the weight gain and food efficiency ratio:

FER = weight gain (g)/food intake (g) × 100
(1)

### 2.5. Chemical and Biochemical Analyses

At the end of the experiment, the animals were sacrificed under a CO_2_ atmosphere. Incision of the abdominal and thoracic cavities was then performed for blood collection.

Plasma and the erythrocyte mass were separated for determination of Zn. The right femur was also removed for further analysis.

Atomic absorption spectrophotometry was used for determination of Zn in plasma after dilution in ultrapure water, and also the erythrocyte mass was determined. In the right femur, Zn analysis was performed after digestion in a nitropercloric: ultrapure water mixture (3:1 v/v) and appropriate dilutions with ultrapure water. In the femur calcium and magnesium were also quantified, adding a solution of strontium chloride hexahydrate (SrCl2·6H2O) after digestion and before the reading [16].

The concentration of hemoglobin in the erythrocyte mass was also quantified, and the erythrocyte Zn was expressed in μg Zn/g Hb. Hemoglobin was determined by the cyanide methemoglobin method, using the kit for colorimetric diagnosis *in vitro* of Bioclin (Belo Horizonte, MG, Brazil). The bones were weighed using a digital analytical balance (Ohaus^®^, Pine Brook, NJ, USA), with accuracy of 0.0001 g. The length, width and outer thickness of the femur were measured using a caliper.

### 2.6. Mineral Retention

Mineral retention of Zn, calcium (Ca) and magnesium (Mg), considering the quantity of mineral deposited in the femur and total quantity of mineral ingested, was determined by means of the diets consumed during the experiment, according to the following equation:

Mineral Retention = mg Mineral (femur) × 100/mg Total Mineral Ingested
(2)

### 2.7. Experimental Design and Analysis of the Data

A completely randomized design was used, in a 2 × 2 factorial design (source *versus* dose), with 10 replicates (animals). Data was analyzed by analysis of variance at 5% probability using the Statistical Analysis System software (SAS), version 8.0, licensed to the Federal University of Viçosa.

## 3. Results and Discussion

### 3.1. Concentration of Zinc in the Experimental Diets

In analysis of the experimental diets, it was found that those planned to receive 30 mg Zn/kg showed 27.93 ± 4.3 mg Zn/kg (test diet) and 31.26 ± 4.23 mg Zn/kg (control diet), not differing from each other (*p* > 0.05). The diets of 15 mg Zn/kg provided 17.45 ± 1.48 mg Zn/kg (test diet) and 12.48 ± 2.25 mg Zn/kg (control diet), and also did not differ (*p* > 0.05).

### 3.2. Biological Assay

There was no effect of the source x dose interaction in any of the variables analyzed (*p* > 0.05), nor the dose for these variables (*p* > 0.05), *i.e.*, there was no significant difference between weight gain (WG), food intake (FI), Zn ingestion (ZnI) and food efficiency ratio (FER) when comparing two different doses (15 and 30 mg Zn/kg) (data not shown). However, the source factor presented significant difference for WG (*p* = 0.0025) and FER (*p* = 0.0083) when analyzed separately, *i.e.*, there was no difference in WG and FER in the animals when the source of Zn was different, and that the control group had the highest average WG (147.83 ± 22.31 g) and FER (18.71% ± 3.69%) when compared to the test group (Table 2).

**Table 2:** Weight gain (WG), food intake (FI), Zn ingestion (ZnI) and feed efficiency ratio (FER) for different sources of Zn: fortified rice (Ultra Rice^®^) or zinc carbonate (ZnCO_3_).

Source	WG (g)	FI (g)	ZnI (mg)	FER (%)
ZnCO_3_	147.83 ± 22.31 ^a^	819.83 ± 60.34 ^a^	17.61 ± 8.18 ^a^	18.71 ± 3.69 ^a^
UR^®^	124.12 ± 23.53 ^b^	798.80 ± 60.68 ^a^	18.49 ± 5.11 ^a^	15.39 ± 3.87 ^b^

Results expressed in mean ± standard deviation of variables of rats after 42 days of diet containing 50% or 100% of zinc recommendations for animals, from zinc oxide; test diet = fortified rice or control diet = zinc carbonate, (*n* = 10 rats/group). Means followed by the same letters in columns do not differ at 5% probability by analysis of variance (ANOVA).

It was observed that food intake did not differ between groups, as with zinc intake. However, animals in the test group which consumed fortified rice showed the lowest mean weight gain and FER. The caloric density of the diets was similar (Table 1), with no differences between the amounts of carbohydrates, proteins and lipids. The UR^®^ constitutes the only different ingredient between diets, present only in those destined for animals of the test groups. Because these showed less weight gain and lower FER than animals in the control group, whose source of dietary zinc was ZnCO_3_, it is suggested that the zinc oxide present in UR^®^ showed lower performance when compared to the control for this variable in question, *i.e.*, a greater quantity of the test diet should be consumed for its conversion in animal weight.

It is known that Zn is present in all the organs, tissues, fluids and secretions of the body [17]. The human body contains approximately 2 to 2.5 g of Zn, of which 55% are located in the muscles and 30% in the bones [18] which, together with the skin and liver, make up the major pools of this mineral [19]. Thus, the concentration of Zn in the bones makes up, in animals, a good marker for assessing the nutritional status of this mineral [6] and therefore these variables were also analyzed.

As for the variables analyzed in Table 2, no effect was observed for the source x dose interaction, as well as the dose analyzed separately (*p* > 0.05), on the concentration of Zn in the femur (Zn-Femur), femur weight (FW), femur length (FL) and femur width (FW) (data not shown). It was observed, however, that the source of Zn had an effect on Zn-Femur and FW, so that the control group, zinc carbonate, had the highest average of Zn-Femur (0.11 ± 0.02), while the test group, zinc oxide, had the highest average FW (0.89 ± 0.11) (Table 3). The result was unexpected, since animals of the test group presented significantly heavier bones, however, the concentration of Zn in the bones was significantly lower than that of the control group. This means that there are other factors besides the content of zinc that affects the bone’s weight.

**Table 3:** Concentration of Zn in femur (Zn-Femur), femur weight (FW), femur length (FL) and femur width (FW) for different sources of Zn: fortified rice (Ultra Rice^®^) or ZnCO_3_.

Source	Zn-Femur (mg/g)	FW (g)	FL (cm)	FW (cm)
ZnCO_3_	0.11 ± 0.02 ^a^	0.80 ± 0.13 ^b^	3.14 ± 0.07 ^a^	0.30 ± 0.01 ^a^
UR^®^	0.09 ± 0.01 ^b^	0.89 ± 0.11 ^a^	3.11 ± 0.13 ^a^	0.30 ± 0.01 ^a^

Results expressed in mean ± standard deviation of variables of rats after 42 days of diet containing 50% or 100% of zinc recommendations for animals, from zinc oxide; test diet = fortified rice or control diet = zinc carbonate, (*n* = 10 rats/group). Means followed by the same letters in columns do not differ at 5% probability by analysis of variance (ANOVA).

It is known that approximately 10% to 20% of Zn in the blood is in the plasma; the rest is in the erythrocytes. Therefore, the concentrations of Zn in the plasma and erythrocytes are also good markers for evaluating the nutritional status of the mineral and can be used in both human and animal studies [20].

No significant differences were found in the plasma zinc (ZnPlas) and erythrocyte zinc concentrations (ZnEryt) between groups, regardless of the dose (data not shown) or the source, indicating that both showed efficiency in the retention of Zn in the plasma and erythrocytes (Table 4).

**Table 4:** Concentration of plasma Zn (ZnPlas) and erythocyte Zn (ZnEryt) for different sources of Zn: fortified rice (Ultra Rice^®^) or ZnCO_3_.

Source	ZnPlas (μg/mL)	ZnEryt (μg/gHb)
UR^®^	77.71 ± 25.17 ^a^	32.09 ± 12.92 ^a^
ZnCO_3_	98.05 ± 37.66 ^a^	29.68 ± 17.79 ^a^

Results expressed in mean ± standard deviation of variables of rats after 42 days of diet containing 50% or 100% of zinc recommendations for animals, from zinc oxide; test diet = fortified rice or control diet = zinc carbonate, (*n* = 10 rats/group). Means followed by the same letters in columns do not differ at 5% probability by analysis of variance (ANOVA).

In animal models, growth and incorporation of Zn in the femur in rats, quails and pigs have been used to assess the bioavailability of Zn [6]. Alkaline phosphatase is an enzyme produced mainly by osteoblasts, whose main function is to promote the deposition of calcium (Ca) in the bone shaft [21,22]. Alkaline phosphatase activity decreases rapidly in animals fed Zn-deficient diets [18], where the magnesium (Mg) ion is also a strong activator of the enzyme [23]. As a consequence, smaller amounts of Zn and Mg provoke lower retention of Ca.

Aiming to evaluate this interaction between Zn, Ca and Mg *in vivo*, retention of these minerals in the femur was also evaluated in this study. In this case, the source × dose interaction was significant in relation to the retention of Zn in the femur (*p* = 0.0088) (data not shown), leading to the need to break down the components of this interaction (Table 5).

**Table 5:** Retention of zinc (RZn-femur), calcium (RCa-femur) and magnesium in femur (RMg-femur) for different sources of Zn: fortified rice (Ultra Rice^®^) or ZnCO_3_.

Source × Dose	RZn-Femur (mg/100 g)	RCa-Femur (mg/100 g)	RMg-Femur (mg/100 g)
C30	1.06 ± 0.25 ^a^	4.35 ± 1.56 ^a^	0.35 ± 0.08 ^a^
R30	0.63 ± 0.12 ^b^	3.40 ± 0.93 ^a^	0.33 ± 0.10 ^a^
C15	0.54 ± 0.23 ^c^	4.12 ± 1.12 ^a^	0.34 ± 0.10 ^a^
R15	0.46 ± 0.15 ^c^	3.52 ± 1.25 ^a^	0.32 ± 0.07 ^a^

Results expressed in mean ± standard deviation of variables of rats after 42 days of diet containing 50% or 100% of zinc recommendations for animals, from zinc oxide; test diet = fortified rice, or control diet = zinc carbonate, (*n* = 10 rats/group). Means followed by the same letters in columns do not differ at 5% probability by analysis of variance (ANOVA); C30: Source of Zn:Zn carbonate, Zn 100%; C15: Source of Zn:Zn carbonate, Zn 50%; R30: Source of Zn:Fortified rice, Zn 100%; R15: Source of Zn:fortified rice, Zn 50%.

No differences were found regarding retention of Ca and Mg in the femur for any of the sources or doses evaluated (*p* > 0.05). However, it was observed that the retention of Zn in the femur was higher (*p* < 0.001) in animals of the control group (ZnCO_3_) when they consumed diets with higher concentrations of this mineral (30 mg of Zn/kg), followed by those who consumed diets containing 30 mg Zn/kg from the rice fortified with zinc oxide. Therefore, for this variable the greater dose of zinc in the diet appears to have influenced the greater retention of this mineral in the bone. It is also noteworthy that animals consuming diets containing 15 mg Zn/kg did not differ with regards to retention of zinc in the femur, independent of the source used (zinc oxide or zinc carbonate), indicating that the UR^®^ was as effective as the control even at the lowest dose.

Although zinc oxide is the most widely used compound for fortification of foods with zinc, some authors suggest that this compound should be avoided due to its low solubility, a factor that affects bioavailability of the mineral [24,25]. However, the use of zinc oxide presents advantages compared to the other compounds, like zinc sulfate, including its greater stability, lower cost and the fact that it does not significantly alter the sensory characteristics of the food to which it is added. Zinc sulfate is the second most commonly used salt for fortification of foods, and presents greater stability than zinc oxide [14].

The generalized use of food fortification and supplementation as strategies for control of micronutrient deficiency makes the interactions between micronutrients a subject of special nutritional relevance, since they may compete by the same absorption sites, thus reducing their bioavailability [14].

The fortified rice in this study presents an iron: zinc ratio of 7.2:1, therefore this does not appear to be the cause of the findings in relation to the lower concentration and retention of zinc in the bone. However, a study demonstrated a negative effect on zinc absorption when iron was added at an iron: zinc ratio of 25:1 to food [6]. Still, the authors observed that when offered as an aqueous solution, similar to the intake of iron supplements, zinc absorption was reduced by iron in a dose-dependent manner. However, when iron was added to solid food or infant formulas, no effect on zinc absorption was observed in adults [6,26].

Similarly, in a review of studies that tested the effect of iron on zinc absorption it was found that iron reduced zinc absorption when added to water, but in this review, only one study showed a negative effect of iron on the bioavailability of zinc when added to solid foods [27].

In the present study, to obtain rice by means of the Ultra Rice^®^ technology, iron and zinc were added to rice flour which was transformed into a solid matrix and subjected to the extrusion process. Therefore, the negative effect of iron in this product on the bioavailability of zinc is unlikely.

Concentrations of fortification agents should be carefully evaluated, especially in relation to minerals that may be harmful to humans at certain intake levels. The decision regarding the dose of the fortification agent to be used should be based on knowledge of the total amount of the fortified food that can be consumed and the amount of the mineral in other foods in which it is also present. When the variability of consumption is high, it is better to be more conservative with regards to the quantities of the mineral to be added to foods so as to avoid the risk of excessive intake in a proportion of the population which regularly consumes fortified products in large quantities [14].

Wheat flour, for example, can be fortified with at least 100 mg zinc/kg without adverse effects on sensory properties and acceptability of fortified products [28]. Even lower levels of zinc addition, ranging from 20 to 30 mg/kg, would be beneficial to aid in meeting the nutritional recommendations [29]. A further study suggested that zinc fortification should be performed in an amount equivalent to 20–50 mg/kg of corn flour [14]. The fortified rice used in this study presented a zinc content of 42 mg Zn/kg of rice, encountered within the fortification range indicated.

## 4. Conclusions

The control diet, whose source of Zn was ZnCO_3_, showed the best results for the parameters of weight gain, food efficiency coefficient, and concentration and retention of Zn in the femur compared to the test diet, whose source of Zn was zinc oxide added to the UR^®^. However, no significant differences were encountered for food intake, length and thickness of the femur, plasma Zn and erythrocyte Zn, indicating that Zn present in the test diet showed good bioavailability when compared to the control.

The low solubility of zinc oxide, the fortification agent used in UR^®^, may have been the main factor that contributed to the results encountered. However, this salt has been widely used due to its low cost and greater stability.
